# Smartphone Apps Targeting Physical Activity in People With Rheumatoid Arthritis: Systematic Quality Appraisal and Content Analysis

**DOI:** 10.2196/18495

**Published:** 2020-07-21

**Authors:** Lindsay M Bearne, Mandeep Sekhon, Rebecca Grainger, Anthony La, Mehrdad Shamali, Aliya Amirova, Emma L Godfrey, Claire M White

**Affiliations:** 1 Department of Population Health Sciences, School of Population Health and Environmental Sciences Faculty of Life Sciences & Medicine King's College London London United Kingdom; 2 University of Otago Wellington New Zealand

**Keywords:** rheumatoid arthritis, physical activity, exercise, mobile applications, behavior change techniques, mobile phone

## Abstract

**Background:**

Rheumatoid arthritis (RA) is a disabling, inflammatory joint condition affecting 0.5%-1% of the global population. Physical activity (PA) and exercise are recommended for people with RA, but uptake and adherence tend to be low. Smartphone apps could assist people with RA to achieve PA recommendations. However, it is not known whether high quality, evidence-informed PA apps that include behavior change techniques (BCTs) previously identified as effective for PA adherence are available for people with RA.

**Objective:**

This study aims to systematically identify apps that include goals to facilitate PA for adults with RA and assess app quality and content for the inclusion of relevant BCTs against recommendations for cardiorespiratory, resistance, flexibility, and neuromotor PA and exercise.

**Methods:**

A systematic search of the Apple App Store and Google Play Store in the United Kingdom was conducted to identify English language apps that promote PA for adults with RA. Two researchers independently assessed app quality (mobile app rating scale [MARS]; range 0-5) and content (BCT Taxonomy version 1, World Health Organization, the American College of Sports Medicine, and the European League against Rheumatism recommendations for PA). The completeness of reporting of PA prescription was evaluated using a modified version of the Consensus on Exercise Reporting Template (CERT; range 0-14).

**Results:**

A total of 14,047 apps were identified. Following deduplication, 2737 apps were screened for eligibility; 6 apps were downloaded (2 on the Apple App Store and 4 on the Google Play Store), yielding 4 unique apps. App quality varied (MARS score 2.25-4.17). Only 1 app was congruent with all aspects of the PA recommendations. All apps completely or partially recommended flexibility and resistance exercises, 3 apps completely or partially advised some form of neuromotor exercise, but only 2 offered full or partial guidance on cardiorespiratory exercise. Completeness of exercise reporting was mixed (CERT scores 7-14 points) and 3-7 BCTs were identified. Two BCTs were common to all apps (information about health consequences and instruction on how to perform behavior). Higher quality apps included a greater number of BCTs and were more closely aligned to PA guidance. No published trials evaluating the effect of the included apps were identified.

**Conclusions:**

This review identifies 4 PA apps of mixed quality and content for use by people with RA. Higher quality apps were more closely aligned to PA guidance and included a greater number of BCTs. One high-quality app (Rheumatoid Arthritis Information Support and Education) included 7 BCTs and was fully aligned with PA and exercise guidance. The effect of apps on PA adherence should be established before implementation.

## Introduction

### Background

Rheumatoid arthritis (RA) is a disabling, autoimmune inflammatory condition that affects 0.5% to 1% of the global population [1,2]. Evidence-informed guidelines recommend physical activity (PA) and exercise for people with RA tailored to an individual’s baseline PA level, disease activity, and symptoms. PA prescriptions comprise exercise type (ie, cardiorespiratory, resistance, flexibility, or neuromotor training), the number of sets/repetitions, load and/or intensity, recovery time/method of progression, and frequency and duration of exercise sessions [3-7].

However, people with RA tend not to meet the recommended levels of PA [8,9], and there are several barriers that can make changing PA behavior challenging for people with RA without appropriate support and guidance [8-11].

Interventions that target the factors that influence adherence to PA using behavior change techniques (BCTs; ie, strategies that help an individual change their behavior) can improve PA levels and health outcomes [12-14]. Many people with RA would like help to increase their PA from health care professionals [9], but interventions can be difficult to implement due to lack of time, resources, and/or the limited number of appropriately trained health care professionals [15-17]. Consequently, novel methods of delivering interventions that can be tailored for people with RA are needed to support adherence to PA.

With the rapid increase in the availability of mobile apps [18], the development and use of high-quality apps may be a promising approach to support people with RA to reach evidence-informed PA recommendations. However, appraisals of apps for RA symptom monitoring and self-management, including PA, suggested that app quality and content were heterogeneous and they did not consistently provide evidence-based management strategies or include validated symptom measures [19-22]. Apps were seldom developed in collaboration with people with RA or clinicians, and older adults often found them difficult to use [21-23].

The effects of digital interventions on PA adherence in people with RA are unclear. A systematic review of randomized controlled trials (4 trials; n=492 participants) found limited evidence of an effect of interactive digital interventions (ie, interactive information and communication technologies to support behavior change, such as online fora) on PA adherence in people with RA or juvenile idiopathic arthritis [24]. Trials included 3 to 9 BCTs [24]. No quality rating of the interactive digital interventions using standardized measures was conducted. Thus, the systematic identification and evaluation of apps that can support adherence to PA and evidence to support the effectiveness of these apps are required.

Recommendations suggest that the safety, quality, and content of self-management apps (including PA apps) for people with RA should be considered during all stages of development, evaluation, and implementation [25]. Features such as engagement, esthetics, functionality, and information quality should be assessed using reliable tools, such as the mobile app rating scale (MARS) [26]. Content should be evaluated for (1) BCTs using a recognized framework (eg, BCT Taxonomy version 1, BCTT v1) [27], (2) congruence with evidence-informed recommendations on PA (eg, World Health Organization and the European League against Rheumatism) [3,5] and exercise (eg, American College of Sports Medicine) [4,6,28], and (3) described using standardized reporting formats such as the Consensus on Exercise Reporting Template (CERT) [7,29].

### Objectives

This study aims to systematically identify and evaluate the quality and content of publicly available mobile apps aiming to support the uptake of and adherence to PA in people with RA.

## Methods

### Protocol and Registration

The review protocol was developed a priori by a team consisting of physiotherapists, rheumatologists, and health psychologists with experience in conducting systematic reviews, evaluating mobile health apps, PA, and behavior change interventions. The protocol was not eligible for registration on the International Prospective Register of Systematic Reviews (PROSPERO) as PROSPERO does not register reviews of apps.

### Data Sources

Where possible, this review followed the Preferred Reporting Items for Systematic Review and Meta-Analyses (PRISMA) guidelines for reporting systematic reviews [30].

Systematic individual searches of the Google Play (Samsung Galaxy s8 operating G950FXXS4DSD3/ G950FOXM4DSBA/G950FXXS4DSD3 software with Android version 9) and Apple App Store (iOS 12.3.1 software operating on iPhone 7) were conducted on June 19 and 20, 2019.

Key search terms for RA, PA, and exercise were identified from the literature [22,24] and refined by all coauthors. Search terms were used in isolation and combination to search for all relevant apps (Table 1).

To ensure that all potentially relevant apps were captured, searches for *rheumatoid arthritis* and *arthritis* were also conducted in the United Kingdom National Health Service app library and the Apple App Store in New Zealand, Australia, Canada, and United States using the fnd website [31].

**Table 1:** Key search terms used for identifying mobile apps targeting physical activity in people with rheumatoid arthritis.

Search terms	Rheumatic	Arthritis	RA^a^	Rheumatoid arthritis	Inflammatory arthritis
Physical activity	Rheumatic physical activity	Arthritis physical activity	RA physical activity	Rheumatoid arthritis physical activity	Inflammatory arthritis physical activity
Exercise	Rheumatic exercise	Arthritis exercise	RA exercise	Rheumatoid arthritis exercise	Inflammatory arthritis exercise
Walking	Rheumatic walking	Arthritis walking	RA walking	Rheumatoid arthritis walking	Inflammatory arthritis walking
Strength	Rheumatic strength	Arthritis strength	RA strength	Rheumatoid arthritis strength	Inflammatory arthritis strength
Fitness	Rheumatic fitness	Arthritis fitness	RA fitness	Rheumatoid arthritis fitness	Inflammatory arthritis fitness
Training	Rheumatic training	Arthritis training	RA training	Rheumatoid arthritis training	Inflammatory arthritis training
Running	Rheumatic running	Arthritis running	RA running	Rheumatoid arthritis running	Inflammatory arthritis running

^a^RA: rheumatoid arthritis.

### App Selection

#### Eligibility Criteria

Deduplication based on app store title and description (provided in the *read more* section in the app) was conducted by 2 independent reviewers (R1 and R2), and potentially eligible apps were downloaded on one or both devices, where possible. The information section for each potentially suitable app was reviewed against the eligibility criteria. Inclusion criteria comprised (1) a smartphone-based app available in at least one app store; (2) targeted at adults (≥18 years) with RA as users specifically, (3) focused on promoting uptake and adherence to PA or exercise, and (4) available in English. Apps were excluded if they (1) targeted people with a condition other than RA, (2) were solely for use by health care practitioners, and (3) were specific clinic, congress/conference, or product apps. No cost restrictions were applied and full app content was purchased, if required.

### Data Items and Extraction

Data extraction was conducted by 2 independent reviewers (R1 and R2) using a data extraction tool developed a priori. Each app was used for at least 10 min. The following app characteristics were extracted: app name, platform, version, developer, stakeholder involvement in app development, size, star rating, number of installs, privacy policy statements, and medical product status. The availability of published trials evaluating app efficacy or effectiveness was checked on developer websites and by searching electronic databases (last search in June 2019) using the search strategy described in our systematic review to synthesize the evidence for the effectiveness of mobile apps designed to enhance adherence to PA for people with inflammatory arthritis (PROSPEROCRD42019129341) [32]. In addition, searches in Google, Google Scholar, and PubMed were undertaken using the app name as a search term. The review of search results was stopped after the first 50 irrelevant results.

### Quality Appraisal of Individual Apps

The quality of the included apps was assessed using the simple and reliable MARS [26]. A total of 23 items were rated on a 5-point Likert scale (1=inadequate to 5=excellent) and summarized into 4 categories: engagement (5 items), functionality (4 items), esthetics (3 items), information quality (7 items), and a subjective quality scale (4 items). The mean score for each category and the MARS total score (the mean of the 4 category scores excluding subjective quality scale) was calculated (maximum score=5). Item 19 of the MARS, *evidence base*, was excluded from all calculations because no apps had been studied in clinical trials, as specified by Stoyanov et al [26].

### Content Analysis of Individual Apps

PA and exercise recommendations for people with RA were evaluated (yes, partial, or no) for congruence with evidence-informed guidance for cardiorespiratory, resistance, flexibility, and neuromotor PA [3-6]. This three-point scale reflected the format of the checklists used to assess the fidelity of rehabilitation and exercise intervention delivery [33-35]. Apps were considered to be fully aligned with the guidance if they included details of exercise type, intensity (eg, load, sets, repetitions), frequency, and time/duration of exercise/sessions (*yes*), partially aligned with guidance if they included at least one of these parameters (*partial*) and not aligned with the guidance if they did not include any of these parameters (*no*). Content was assessed against the PA guidance that was most appropriate for the age and exercise experience of the target user for each app.

Guidance indicates that adults (aged 18 to 64 years) should perform ≥150 min of cardiorespiratory exercise at moderate intensity or 75 min of vigorous/high-intensity activity or an equivalent combination per week in bouts ≥10 min. Adults who are novice (ie, unaccustomed to exercise) or intermediate (ie, some experience of exercise) exercisers should also perform between 2 and 4 sets of resistance exercises (8 and 12 repetitions per set) at a moderate/hard intensity (ie, 60% to 70% of one repetition maximum) for each major muscle group on at least two days per week. However, experienced exercisers (ie, engaged in habitual exercise) should work at a hard or very hard intensity (ie, ≥80% one repetition maximum). Exercises to increase or maintain flexibility are advised at least twice per week for a minimum of 10 min. Each stretch should be held to the point of tightness or slight discomfort for 10 to 30 seconds up to a total of 60 seconds of stretching time per exercise. Adults with poor mobility and balance are advised to perform 20 to 30 min of neuromotor exercises (eg, balance, agility, coordination, proprioceptive exercise training, or multifaceted activities such as yoga) on at least two days per week to enhance physical function, balance, and prevent falls [3-5].

Guidelines for older adults (≥65 years) and adults between 50 and 64 years with long-term conditions are similar, although activities should be tailored to health and disease status, baseline fitness, and initially comprise very light or light intensity resistance exercises (ie, 40% to 50% of one repetition maximum) and stretches of a 30- to 60-second duration [3,5,6].

The CERT is a reliable, 16-item (7 domain) specification to evaluate the reporting of exercise interventions (Table 2) [7,29,36]. Each item was rated (yes=1, no=0, or not applicable) and summed to produce a total CERT score. It was not possible to score items 11 or 16; therefore, the maximum possible CERT score was 14 [7,29].

**Table 2:** Abbreviated item description for the Consensus of Exercise Reporting Template.

Item category and item number		Abbreviated item description
**What**		
	1	Description of type of exercise equipment
**Who**		
	2	Description of qualifications/expertise/training of instructor
**How**		
	3	Description of whether exercises are performed individually or in a group
	4	Description of whether exercises are supervised/unsupervised
	5	Description of the measurement/reporting of adherence to exercise
	6	Description of motivational strategies
	7	Decision rules for determining exercise progression and how exercise was progressed
	8	Description of each exercise to enable replication (eg, illustrations, photos)
	9	Description of any home programme component
	10	Description of any nonexercise component
	11	Description of the type/number of adverse events that occurred during exercise
**Where**		
	12	Description of exercise setting
**When, how much**		
	13	Description of exercise intervention and dosage
**Tailoring**		
	14	Description of whether exercises are generic or tailored to the individual
	15	Decision rule for starting level of exercise
**How well**		
	16	Description of whether the exercise is delivered and performed as planned

Two postgraduate physiotherapy students (R1 and R2) independently assessed app quality using the MARS and PA and exercise content for congruence with evidence-informed recommendations and reporting using the CERT. Discrepancies were resolved by discussion and another assessor acted as an arbiter if required. As recommended by the MARS developers, assessors received training on applying the MARS by viewing the MARS training video [26,37] and bespoke training to assess app content for congruence with PA recommendations and completeness of reporting by a member of the CERT development group [7].

Before rating the included apps, the assessors evaluated up to 5 randomly selected apps (identified in the search but previously excluded from the analysis) and discussed their results to ensure an understanding of the MARS, PA and exercise recommendations, and CERT items and processes.

The BCTT v1 is a comprehensive and reliable tool that consists of 93 distinct BCTs that can be used to identify specific *active components* of behavioral interventions [27]. It is used to design and retrospectively evaluate behavioral health interventions, such as PA [38].

Two postgraduate health psychologists (R3 and R4) completed the training for applying the BCTT v1 before independently coding BCTs in the included apps [27]. Discrepancies were resolved by discussion, and another rater acted as an arbiter if required.

### Statistical Analysis

Interrater reliability using an intraclass correlation coefficient (ICC) was calculated using IBM SPSS version 25.0 (two-way random-effects model of absolute agreement between single ratings). Scores <0.5 were considered poor agreement, moderate reliability (0.5-0.75), good reliability (0.75-0.9), and excellent reliability (>0.9) [39].

## Results

### App Selection

The systematic search identified 14,047 apps (UK Google Play Store n=11,750 and Apple App Store n=2297). No further apps were identified from the United Kingdom National Health Service app library and the Apple App Store in the United States, New Zealand, Australia, or Canada.

**Figure 1:**
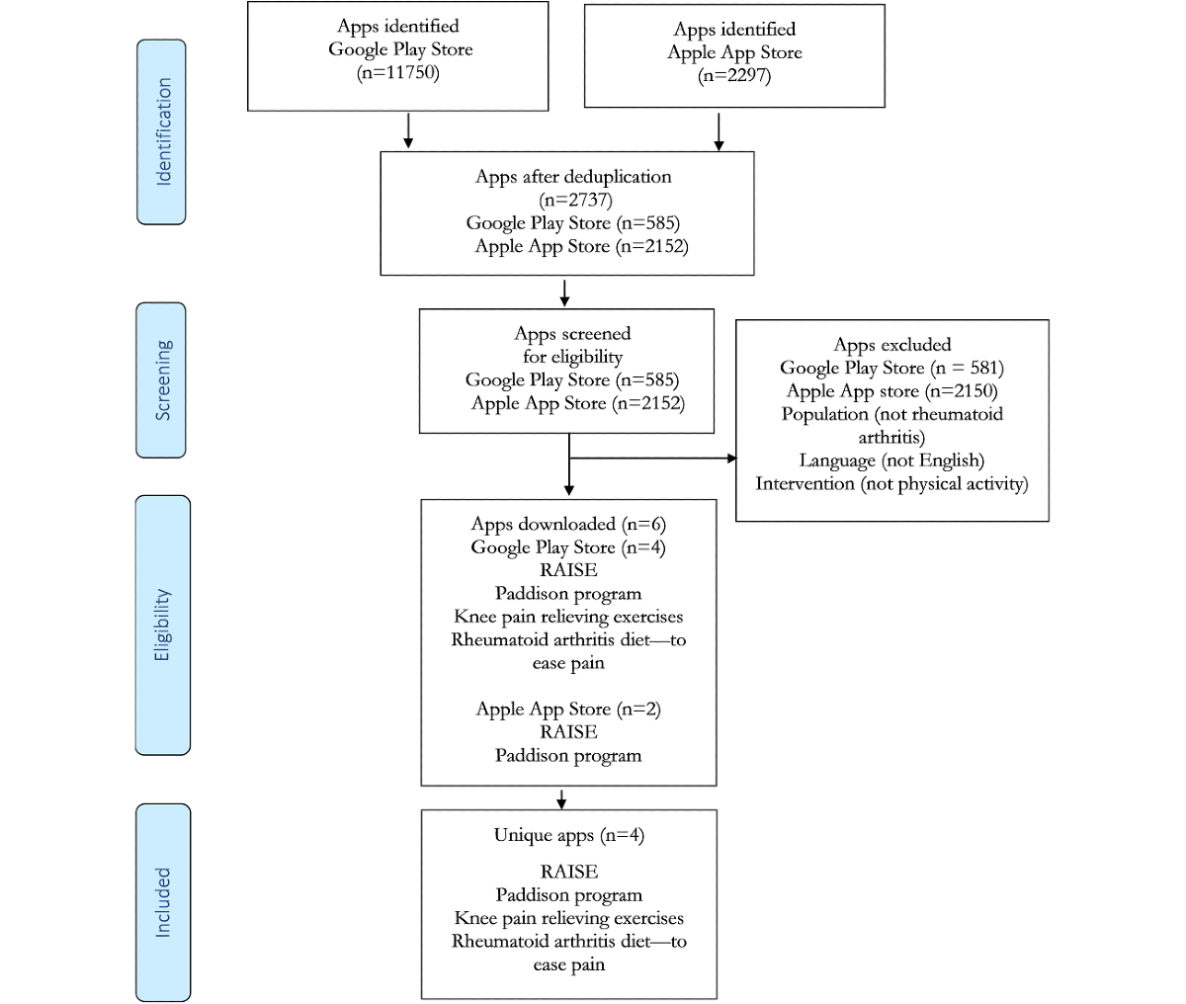
Flow diagram illustrating the process of mobile app selection.

Following deduplication, 2737 app titles and descriptions were screened and 6 apps (Google Play Store n=4 and Apple App Store n=2) met the eligibility criteria, yielding 4 unique apps (Rheumatoid Arthritis Information Support and Education [RAISE], Paddison program, Knee pain relieving exercises, Rheumatoid arthritis diet—to ease pain; Figure 1). Two apps (Knee pain relieving exercises and Rheumatoid arthritis diet—to ease pain) were exclusively available on the Google Play Store, and 2 apps were available on both platforms (RAISE and Paddison program; Figure 1).

### Characteristics of the Included Apps

The characteristics of the included apps are detailed in Table 3. A total of 3 apps were developed by or in conjunction with people with RA (RAISE and Paddison program) or clinicians (RAISE and Knee pain relieving exercises). Apple App store star ratings of included apps ranged from 2 (RAISE on Android) to 5 stars (RAISE on iOS), although the number of ratings was generally low (Knee pain relieving exercises n=140 and RAISE-iOS n=1 ratings). All mobile apps were free to download. The Paddison program app required a payment of US $69 to access the Paddison program resources and daily videos for 12 days (*essential package*). A fee of US $99 provided access to all content, that is, Paddison program resources, 12 daily videos, and additional updated video/website content (*advanced healing package*).

The apps had been installed 500 (RAISE on Android) to 50,000 (Knee pain relieving exercises) times. None of the included apps had a medical product status. No published trials evaluating the effect of the included apps were identified.

**Table 3:** Characteristics and quality rating of mobile apps targeting physical activity in people with rheumatoid arthritis.

App	Knee pain relieving exercises	Rheumatoid arthritis diet—to ease pain	RAISE^a^			Paddison program	
A platform	Android^b^	Android^b^	Android	iOS	Android		iOS
Developer	Dr Kavin Khatri	TP Topics	Publicis Dublin	Publicis Dublin	Paddison program		Paddison program
Stakeholder involvement in app development	Clinician	N/A^c^	Patient organization and clinicians	Patient organization and clinicians	Patient representative		Patient representative
Version	1.0.0	1.1.2	1.1.2	1.0.0	1.2.0		2.1.0
Size, MB^d^	4.84	16.27	10.18	18.90	36.31		48.80
Star rating (number of ratings)	4.0 (140)	3.0 (3)	2.0 (2)	5.0 (1)	4.4 (8)		—^e^
Number of installations	>50,000	>1000	>500	—	>1000		—
Privacy policy statement (yes/no)	Yes	Yes	Yes	Yes	Yes		Yes
Medical product status (yes/no)	No	No	No	No	No		No
Peer-reviewed publications (yes/no)	No	No	No	No	No		No
MARS^f^-engagement	2.00	2.40	3.90	3.90	3.70		3.70
MARS-functionality	4.50	3.12	4.38	4.38	3.88		3.88
MARS-esthetics	2.33	3.00	4.33	4.33	3.66		4.00
MARS-information	2.83	2.83	4.08	4.08	3.33		3.33
MARS-subjective	1.88	2.84	3.25	3.25	2.00		2.00
MARS-total	2.92	2.83	4.17	4.17	3.64		3.73

^a^RAISE: Rheumatoid Arthritis Information Support and Education.

^b^Apps not available on the iOS platform.

^c^N/A: not applicable.

^d^MB: megabytes.

^e^Data not available.

^e^MARS: mobile app rating scale.

### Quality Appraisal of the Included Apps

The MARS total score ranged from 2.83 (Rheumatoid arthritis diet—to ease pain) to 4.17 (RAISE), indicating variation in app quality (Table 3). The esthetics category showed the greatest variability (2.30 for Knee pain relieving exercises to 4.33 for RAISE). A total of 3 of the 4 apps (Knee pain relieving exercises, Rheumatoid arthritis diet—to ease pain, and RAISE) scored their highest MARS score for the functionality category and their lowest MARS score for the engagement category. The Paddison program achieved its highest score for esthetics on the iOS platform but not the Android platform. This is because some items (eg, logos) were distorted or not visible on the Android platform. The Paddison program achieved its lowest score for the information quality category on both platforms.

Only 1 app (RAISE) was rated >4 out of 5 for the MARS total score and achieved the highest score in all categories (ie, engagement, esthetics, information, subjective) except functionality. The Knee pain relieving exercise app scored highest for functionality.

The interrater reliability for the MARS indicated excellent agreement for all subscales: engagement (ICC 0.96; 95% CI 0.71-0.99), functionality (ICC 0.92; 95% CI 0.01-0.99), esthetics (ICC 1.00 absolute agreement), information (ICC 0.95; 95% CI 0.64-0.99), subjective (ICC 0.98; 95% CI 0.83-0.99), and the MARS total score (ICC 0.99; 95% CI 0.92-0.99).

### Content Analysis of the Included Apps

The content of the included apps is summarized in Tables 4 to 6. All apps completely or partially recommended flexibility and resistance exercises. Three apps completely or partially advised some form of neuromotor exercise (Rheumatoid arthritis diet—to ease pain, RAISE, and Paddison program). Two apps offered full or partial guidance on cardiorespiratory exercise (Rheumatoid arthritis diet—to ease pain and RAISE; Table 4). 

Only 1 app (RAISE) was congruent with all aspects of the evidence-informed PA recommendations. However, the RAISE app recommended 30 min of cardiorespiratory exercise daily (equivalent to 210 min per week), which is in excess of the minimum weekly PA recommendations.

The Rheumatoid arthritis diet—to ease pain app was primarily focused on providing dietary advice but also recommended all types of PA. It was rated as partially adhering to the guidance because PA dosages were not specified.

**Table 4:** Congruence with evidence-informed recommendations in mobile apps targeting physical activity in people with rheumatoid arthritis.

Physical activity and exercise recommendations	Knee pain relieving exercises	Rheumatoid arthritis diet—to ease pain	RAISE^a^	Paddison program
Cardiorespiratory exercise	No	Partial	Yes	No
Resistance exercise	Partial	Partial	Yes	Partial
Flexibility exercise	Partial	Partial	Yes	No
Neuromotor exercise	No	Partial	Yes	Partial

^a^RAISE: Rheumatoid Arthritis Information Support and Education.

Completeness of exercise reporting in apps using CERT is summarized in Table 5. All apps offered information on the (1) exercise format (individual, unsupervised, or home-based), (2) the potential positive benefits of exercise as a motivational strategy, and (3) nonexercise advice (eg, lifestyle or medication). All apps specified some form of guidance for exercise progression, although the parameters for exercise progression did not always align with guidance. All apps, except Rheumatoid arthritis diet—to ease pain, provided details of (1) the exercise equipment required; (2) the qualification/training of the exercise instructor; and (3) descriptions, adaptations, and dosage of the exercises. Two apps offered decision rules to help users determine an initial exercise dose (RAISE and Paddison program). One app (RAISE) offered the option to document exercise adherence.

The RAISE app reported all types of PA completely and achieved the maximum possible score (14 out of 14). The Rheumatoid arthritis diet—to ease the pain app achieved the lowest CERT score (7 out of 14), predominantly because it did not explicitly report PA dosages. Interrater reliability for CERT scoring was good (ICC 0.796; 95% CI 0.806-0.933).

**Table 5:** Physical activity and exercise reporting in accordance with the Consensus of Exercise Reporting Template in mobile apps targeting physical activity in people with rheumatoid arthritis.

Items and item number			Knee pain relieving exercises		Rheumatoid arthritis diet—to ease pain		RAISE^a^		Paddison program
**What**									
	1	Yes		No		Yes		Yes	
**Who**									
	2	Yes		No		Yes		Yes	
**How**									
	3	Yes		Yes		Yes		Yes	
	4	Yes		Yes		Yes		Yes	
	5	No		No		Yes		No	
	6	Yes		Yes		Yes		Yes	
	7	Yes		Yes		Yes		Yes	
	8	Yes		No		Yes		Yes	
	9	Yes		No		Yes		Yes	
	10	Yes		Yes		Yes		Yes	
	11	N/A^b^		N/A		N/A		N/A	
**Where**									
	12	Yes		Yes		Yes		Yes	
**When and how much**									
	13	Yes		No		Yes		No	
**Tailoring**									
	14	Yes; tailored		Yes; generic		Yes; tailored		Yes; tailored	
	15	No		No		Yes		Yes	
**How well**									
	16	N/A		N/A		N/A		N/A	
Total Score			12		7		14		12

^a^RAISE: Rheumatoid Arthritis Information Support and Education.

^b^N/A: not applicable.

The apps included between 3 (Rheumatoid arthritis diet—to ease pain) and 7 (RAISE and Paddison program) BCTs. Two BCTs were identified in all apps (instructions on how to perform behavior and information about health consequences; Table 6). Credible source was present in all apps but the Paddison program. All apps included demonstration of behavior apart from the Rheumatoid arthritis diet—to ease pain app. The RAISE and Paddison program apps both included 7 BCTs. Five of these BCTs were common to both apps (instruction on how to perform behavior, information about health consequences, demonstration of behavior, goal setting, and social comparison). Only the RAISE app included self-monitoring of behavior. The interrater reliability for BCTs was good (ICC 0.874; 95% CI 0.799-0.921).

**Table 6:** Behavior change techniques included in mobile apps targeting physical activity in people with rheumatoid arthritis.

Behavior change techniques	Knee pain relieving exercises	Rheumatoid arthritis diet—to ease pain	RAISE^a^	Paddison program
Instruction on how to perform behavior	Yes	Yes	Yes	Yes
Information about health consequences	Yes	Yes	Yes	Yes
Demonstration of behavior	Yes	No	Yes	Yes
Credible source	Yes	Yes	Yes	No
Goal setting behavior	No	No	Yes	Yes
Social comparison	No	No	Yes	Yes
Graded task	Yes	No	No	Yes
Self-monitoring behavior	No	No	Yes	No
Generalization of target behavior	No	No	No	Yes
Framing/reframing	No	No	No	Yes
Total number	5	3	7	7

^a^RAISE: Rheumatoid Arthritis Information Support and Education.

## Discussion

### Principal Findings

This is the first systematic identification, quality appraisal, and content analysis of widely available PA and exercise apps for people with RA. Up to June 20, 2019, there were only 4 unique apps that met our inclusion criteria available on iOS and/or Android platforms. The quality and content of the included apps varied considerably and did not always align with PA recommendations. Notably, higher-quality apps tended to include a greater number of BCTs and most closely aligned to PA recommendations. The highest quality app (RAISE) was the only app to explicitly report PA prescriptions aligned to evidence-informed recommendations for people with RA and embedded the highest number of BCTs.

Despite guidance for the development and evaluation of mobile apps [25,40], the quality ratings of the apps included in our review were mixed. This finding is consistent with reviews of publicly available rheumatology self-management apps [20-22] and apps targeting PA and exercise in the general population [41,42]. For example, Simoes et al [43] identified 51 moderate-quality PA apps for use by the general population (MARS total score 3.16 to 4.41) with the functionality and esthetics domains scoring most highly. This is broadly similar to our findings and suggests that the included apps were intuitive, logical to follow, easy to learn and navigate, which is particularly important for people with RA who may have fatigue or hand and wrist disability [44,45]. However, the apps achieved lower ratings for information quality and engagement and to optimize utility, app content should be high quality, interesting, simple to understand, and have the option to be tailored with user data [25].

The RAISE app was the only app that fully and explicitly reported PA prescription that aligned to all evidence-informed recommendations for people with RA. This may be because the RAISE app was the only app to be developed in conjunction with both clinicians and people with RA; thus, concordance with evidence-based guidelines and the acceptability and user experience of people with RA were likely to be considered from inception.

Even though the RAISE app was rated as congruent with all PA guidance, it recommended a weekly duration of cardiorespiratory PA in excess of the minimum dosage stated in the guidelines, which may be inappropriate for novice exercisers with RA. Interestingly, the RAISE app had the lowest number of installs and number of user ratings, suggesting that it is not widely used by people with RA. This may be because people with RA were unaware of the app or because they found the PA recommendations to be too ambitious and unacceptable.

The other apps did not completely align with PA recommendations, similar to other research [41,42]. Common reasons for apps not completely adhering to the PA guidance were the lack of or incorrect specific PA dosages (eg, sets, repetitions). For example, the Rheumatoid arthritis diet—to ease pain app recommended all PA types but did not provide specific PA dosages so was only partially congruent with the guidance. This may be because the primary focus of this app was dietary advice.

PA prescription may need to be modified for people with RA [3,4,6] and some apps offered a reduced starting dose or suggested ways to tailor exercises or exercise progression. However, these recommendations did not always align with guidance. Incomplete or unclear PA prescriptions maybe confusing to users who are novice exercisers, and inappropriate prescriptions may impact users’ engagement and adherence with PA and compromise PA effectiveness or safety [7].

All apps included some BCTs that may promote adherence to PA, and the higher-quality apps included a greater number of BCTs. No app contained more than 7 BCTs, which is similar to the findings of reviews of apps targeting PA in the general population [41,43,46,47]. Although the optimum number of BCTs needed to support PA adherence is not known, a recent systematic review including 8 randomized controlled trials (1018 participants) found that interventions with less than 7 BCTs were most effective at enhancing adherence to exercise in people with persistent musculoskeletal pain [48]. In addition, there was a moderate level of evidence that 5 BCTs (social support [unspecified], goal setting [behavior], instruction of behavior, demonstration of behavior, and behavior practice/rehearsal) supported PA adherence [48]. Two of the higher-quality apps in our review included 3 of these 5 BCTs adding evidence-based integrity, although the effectiveness of these apps has not been investigated.

### Methodological Considerations

The strengths of this review include the comprehensive search of both the UK Google Play Store and Apple App Store. This was complemented by a search of the United Kingdom National Health Service app library and the Apple App Store in the United States, Canada, New Zealand, and Australia. No new apps were identified, suggesting that all English language apps were captured. However, this review only focuses on publicly available apps for use by people with RA, so we may not have captured apps primarily designed for research.

Where possible, we followed rigorous processes that were aligned to PRISMA guidelines [30]. Two reviewers independently screened identified apps for eligibility, extracted data, and rated the quality and content of the apps using standardized tools. Interrater reliability was good or excellent, which lends confidence to our findings. App quality was assessed with the widely used MARS [21,22,26]. However, the MARS rating is subjective, and people with RA may have different perceptions of the app features to our assessors, who did not have RA.

Notably, it is possible to reset star ratings in the iOS app store when new app versions are released. It is not known if the star ratings extracted at the time of our appraisal refer to overall or current app versions ratings. However, all included apps have limited versions, so the impact of this on our findings is likely to be minimal. Finally, no evaluation of the content of the privacy policies of included apps was completed, so we do not know whether the policy adequately protects users’ rights.

This comprehensive review of PA apps for people with RA identified 4 apps of mixed quality and content. Higher quality apps more closely aligned to PA guidance and included a greater number of BCTs previously shown to promote PA. The RAISE app was the highest quality app. Future apps should be rigorously developed with key stakeholders, and include evidence-based PA guidance and BCTs, to optimize their acceptability and impact on PA. Robustly designed research into the effect of apps on PA adherence is crucial before implementation.

## Abbreviations

BCT: behavior change technique
BCTT: Behavior Change Technique Taxonomy
CERT: Consensus on Exercise Reporting Template
ICC: intraclass correlation coefficient
MARS: mobile app rating scale
PA: physical activity
PRISMA: Preferred Reporting Items for Systematic Review and Meta-Analyses
PROSPERO: International Prospective Register of Systematic Reviews
RA: rheumatoid arthritis
RAISE: Rheumatoid Arthritis Information Support and Education
